# An integrated biometric voice and facial features for early detection of Parkinson’s disease

**DOI:** 10.1038/s41531-022-00414-8

**Published:** 2022-10-29

**Authors:** Wee Shin Lim, Shu-I Chiu, Meng-Ciao Wu, Shu-Fen Tsai, Pu-He Wang, Kun-Pei Lin, Yung-Ming Chen, Pei-Ling Peng, Yung-Yaw Chen, Jyh-Shing Roger Jang, Chin-Hsien Lin

**Affiliations:** 1 Department of Computer Science and Information Engineering, National Taiwan University, Taipei, Taiwan; 2 Department of Computer Science, National Chengchi University, Taipei, Taiwan; 3 Department of Electronic Engineering, National Taiwan University, Taipei, Taiwan; 4 Department of Geriatrics, National Taiwan University Hospital, College of Medicine, National Taiwan University, Taipei, Taiwan; 5 Department of Internal Medicine, National Taiwan University Hospital, College of Medicine, National Taiwan University, Taipei, Taiwan; 6 Department of Neurology, National Taiwan University Hospital, College of Medicine, National Taiwan University, Taipei, Taiwan

**Keywords:** Parkinson's disease, Parkinson's disease

## Abstract

Hypomimia and voice changes are soft signs preceding classical motor disability in patients with Parkinson’s disease (PD). We aim to investigate whether an analysis of acoustic and facial expressions with machine-learning algorithms assist early identification of patients with PD. We recruited 371 participants, including a training cohort (112 PD patients during “on” phase, 111 controls) and a validation cohort (74 PD patients during “off” phase, 74 controls). All participants underwent a smartphone-based, simultaneous recording of voice and facial expressions, while reading an article. Nine different machine learning classifiers were applied. We observed that integrated facial and voice features could discriminate early-stage PD patients from controls with an area under the receiver operating characteristic (AUROC) diagnostic value of 0.85. In the validation cohort, the optimal diagnostic value (0.90) maintained. We concluded that integrated biometric features of voice and facial expressions could assist the identification of early-stage PD patients from aged controls.

## Introduction

Parkinson’s disease (PD) is the second most common neurodegenerative disorder causing both motor and non-motor symptoms that result in disability and caregiver burden in aging society^1^. It was estimated that the number of people with PD will rise from 4 million in 2005 by two times to an estimated of 9.3 million in the year 2030^2^. Although dopaminergic treatments and deep brain stimulation could provide symptomatic benefit for some PD motor symptoms, the disease continues to progress with age. Given the likely entry of mechanism-targeted therapies into early human clinical trials, it is crucial to identify susceptible subjects in the premotor or prediagnostic stage of PD, when they are transitioning from normal aging to early-stage PD, to ensure that the proper intervention can mitigate disease progression.

Several clues of soft signs could be detected before the occurrence of the classical motor dysfunction of PD. These early biometric features included reduced facial expressions, voice changes (including reduced speech volume and speech tempo, frequent speech pauses, and shortened speech) and gait pattens with reduced arm-associated movements^3^. However, these mild signs are often ignored and considered normal aging phenomena, which may delay the diagnosis and optimal treatment of PD.

Among these mild biometric features, linguistic changes could be observed as early as 5 years before the PD diagnosis^4^. Additionally, facial bradykinesia, also known as hypomimia or “poker” face, is another early biometric sign of PD. It manifests as a reduction in facial movements, and both the upper and lower face may be affected^5^. Hypomimia is considered a sensitive characteristic of PD, which makes it a potentially promising feature for assisting the early diagnosis of PD^6^.

The current existing digital biomarkers for assisting the diagnosis of PD are focused on motor features, often detected with wearable sensors^7^. Although wearable sensors are reliable, they typically require active participation and are often expensive, which can hinder widespread use in a large population. On the other hand, voice and facial expression analysis, which only requires a webcam or a smartphone with a camera, is a convenient, relatively affordable tool for detecting PD in communities. Researchers or physicians can analyze these biometric features remotely to identify patients that potentially have PD without an in-person interview. This feature may benefit patients that need physical separation from other conditions (e.g., COVID-19) and patients that live in underdeveloped areas without access to a movement disorder specialist.

Although several apps have been developed for the smartphone or smartwatch, those apps mainly focus on detecting the arm swing movement pattern and related motor symptoms^8,9^. A single-domain modality may not be PD-specific, because patients with stroke or arthritis may also manifest reduced arm movement. An integrated multidomain biometric features with automatic analysis and machine-learning algorithms might be more sensitive than a single-feature modality for accurately detecting early-stage PD. Hence, in this study, we aimed to establish a deep learning model that incorporated various biometric features derived from voice and facial expression analyses, which could be used to distinguish patients with early-stage PD from age- and sex-matched healthy controls.

## Results

The demographics of all enrolled patients with PD (*n* = 186) and controls (*n* = 185) are shown in Table 1. The age and sex were comparable between controls and patients with early-stage PD; but those with advanced-stage PD were older than those with early-stage PD and controls (*P* < 0.01). The PD groups had a higher percentage of men compared to controls. Among PD patients, 24 (12.9%) have motor fluctuations (15 patients with Hoehn–Yahr stage 3 and 9 patients were Hoehn–Yahr stage 4) and all these patients with motor fluctuations received the biometric data collection in their “on” phase. The training dataset (i.e., patient data retrieved during the “on” phase, *n* = 112; and age-matched matched controls, *n* = 111) is shown in supplementary Table 1. The validation dataset (i.e., patient data retrieved during the “off” phase, *n* = 74; and age-matched controls, *n* = 74) are shown in supplementary Table 2.

**Table 1:** Clinical characteristics of all study participants.

	Control, *n* = 185	Early stage PD, *n* = 119		Advanced-stage PD, *n* = 67		Controls vs. early PD	Early vs. advanced PD
		H-Y stage 1, *n* = 44	H-Y stage 2, *n* = 75	H-Y stage 3, *n* = 58	H-Y stage 4, *n* = 9	*P* value	*P* value
Sex, male, *N* (%)	84 (45.3)	20 (45.5)	38 (50.6)	41 (70.7)	5 (55.6)	0.06	0.02*
Current age, years	68.5 ± 9.0	64.0 ± 10.0	67.0 ± 8.7	70.4 ± 7.3	74.0 ± 9.6	0.07	<0.01**
Disease duration, years	N.A.	3.9 ± 2.5	7.1 ± 3.2	9.2 ± 3.5	12.5 ± 2.7	N.A.	<0.01**
MDS-UPDRS part III score (on)	N.A.	8.6 ± 3.2	15.7 ± 5.9	20.8 ± 6.5	25.6 ± 7.3	N.A.	<0.01**
MDS-UPDRS part III score(off)	N.A.	12.3 ± 4.3	18.9 ± 6.5	26.3 ± 7.8	30.2 ± 6.9	N.A.	<0.01**
Reading time, seconds	52.3 ± 12.8	52.0 ± 13.4	65.1 ± 28.4	75.3 ± 33.8	71.0 ± 16.3	<0.01**	<0.01**
Phonetic score	96.6 ± 1.7	96.2 ± 2.4	96.0 ± 2.0	94.3 ± 4.2	93.1 ± 5.0	0.89	0.32
Pause percentage, %	13.4 ± 7.3	18.6 ± 8.4	23.6 ± 11.4	30.3 ± 12.9	34.1 ± 13.1	<0.01**	<0.01**
Volume variance	−0.8 ± 10.6	−1.6 ± 12.1	−2.6 ± 10.7	−3.6 ± 9.8	−3.1 ± 12.5	0.13	<0.01**
Pitch variance	16.2 ± 4.5	12.9 ± 4.3	12.8 ± 5.5	12.1 ± 4.0	13.3 ± 5.7	<0.01**	0.37
Average pitch	157.3 ± 32.0	154.3 ± 32.0	151.6 ± 34.0	150.0 ± 33.2	166.7 ± 19.7	0.22	0.95
Mouth angle to eye distances variance (right)	0.004 ± 0.002	0.004 ± 0.003	0.004 ± 0.002	0.004 ± 0.002	0.005 ± 0.003	0.21	0.39
Mouth angle to eye distances variance (left)	0.004 ± 0.002	0.004 ± 0.003	0.005 ± 0.003	0.004 ± 0.002	0.005 ± 0.004	0.23	0.83
Mouth height variance	0.008 ± 0.005	0.007 ± 0.005	0.008 ± 0.004	0.007 ± 0.004	0.008 ± 0.003	0.45	0.64
Mouth width variance	0.007 ± 0.004	0.007 ± 0.005	0.007 ± 0.004	0.006 ± 0.004	0.008 ± 0.005	0.82	0.57
Mouth angle variance	0.872 ± 0.381	0.889 ± 0.482	0.981 ± 0.573	1.045 ± 0.529	1.591 ± 1.074	0.16	0.05
Peri-oral area movement variance (right)	0.027 ± 0.022	0.031 ± 0.038	0.030 ± 0.031	0.029 ± 0.020	0.032 ± 0.029	0.37	0.97
Peri-oral area movement variance (left)	0.030 ± 0.025	0.032 ± 0.031	0.032 ± 0.029	0.033 ± 0.026	0.040 ± 0.041	0.51	0.75
Eye blinking (30% threshold)	0.297 ± 0.265	0.228 ± 0.224	0.210 ± 0.225	0.202 ± 0.214	0.185 ± 0.258	0.01*	0.61
Eye blinking (50% threshold)	0.143 ± 0.169	0.090 ± 0.011	0.086 ± 0.131	0.054 ± 0.091	0.022 ± 0.037	<0.01**	0.02*
Eye blinking (70% threshold)	0.053 ± 0.093	0.046 ± 0.085	0.031 ± 0.069	0.010 ± 0.029	0.002 ± 0.003	0.04*	<0.01**
Eye blinking (90% threshold)	0.005 ± 0.021	0.009 ± 0.029	0.007 ± 0.032	0.001 ± 0.004	0.001 ± 0.003	0.43	0.06

*PD* Parkinson’s disease, *MDS-UPDRS* the Movement Disorder Society-Sponsored Revision of the Unified Parkinson’s Disease Rating Scale, *H-Y stage* Hoehn-Yahr stage.

*P < 0.05; **P < 0.01.

In the voice analysis, patients with PD took longer to read the article, paused more during reading, and their pitch and volume variance were reduced, compared to control participants (Table 1). In the facial expression analysis, among patients with PD, the eye blinking rate was significantly lower than that of controls, but the mouth angle and peri-oral movement variances were not significantly different between groups (Table 1).

### Diagnostic performance based on facial and voice features in the training dataset

**Fig. 1:**
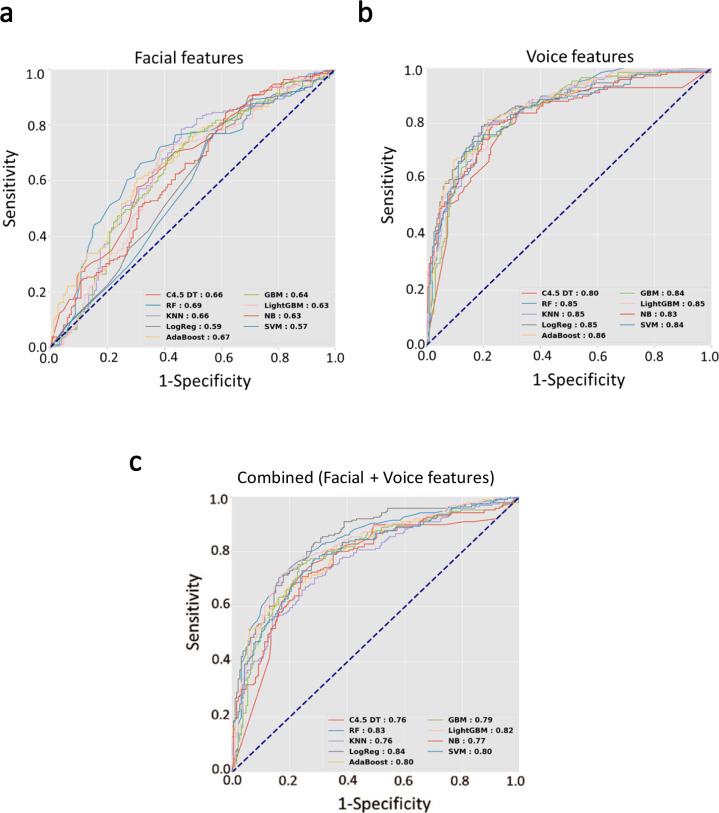
Receiver operating characteristic curves calculated with nine deep-learning classifier models. The models were used to evaluate the predictive value of either **a** facial features alone, **b** voice features alone, or **c** combined both features. The area under the curve indicates the ability to differentiate between the entire patient cohort and the controls, in the training dataset.

In the training dataset, we first examined the performance of the facial expression characteristics alone to differentiate the entire cohort of patients with PD from the controls. Among the various deep-learning classifiers, the random forest classifier showed a diagnostic value of 0.69, when the ROC was analyzed with a discretization and 10-fold cross-validation procedure (Fig. 1a). The diagnostic performance of voice features alone showed that the best classifier was AdaBoost, with an acceptable diagnostic value of 0.86 (Fig. 1b). A combined model with both features of voice and facial expression (age and sex were not included) showed a diagnostic value of 0.84 using the logistics regression classifier (Fig. 1c).

**Fig. 2:**
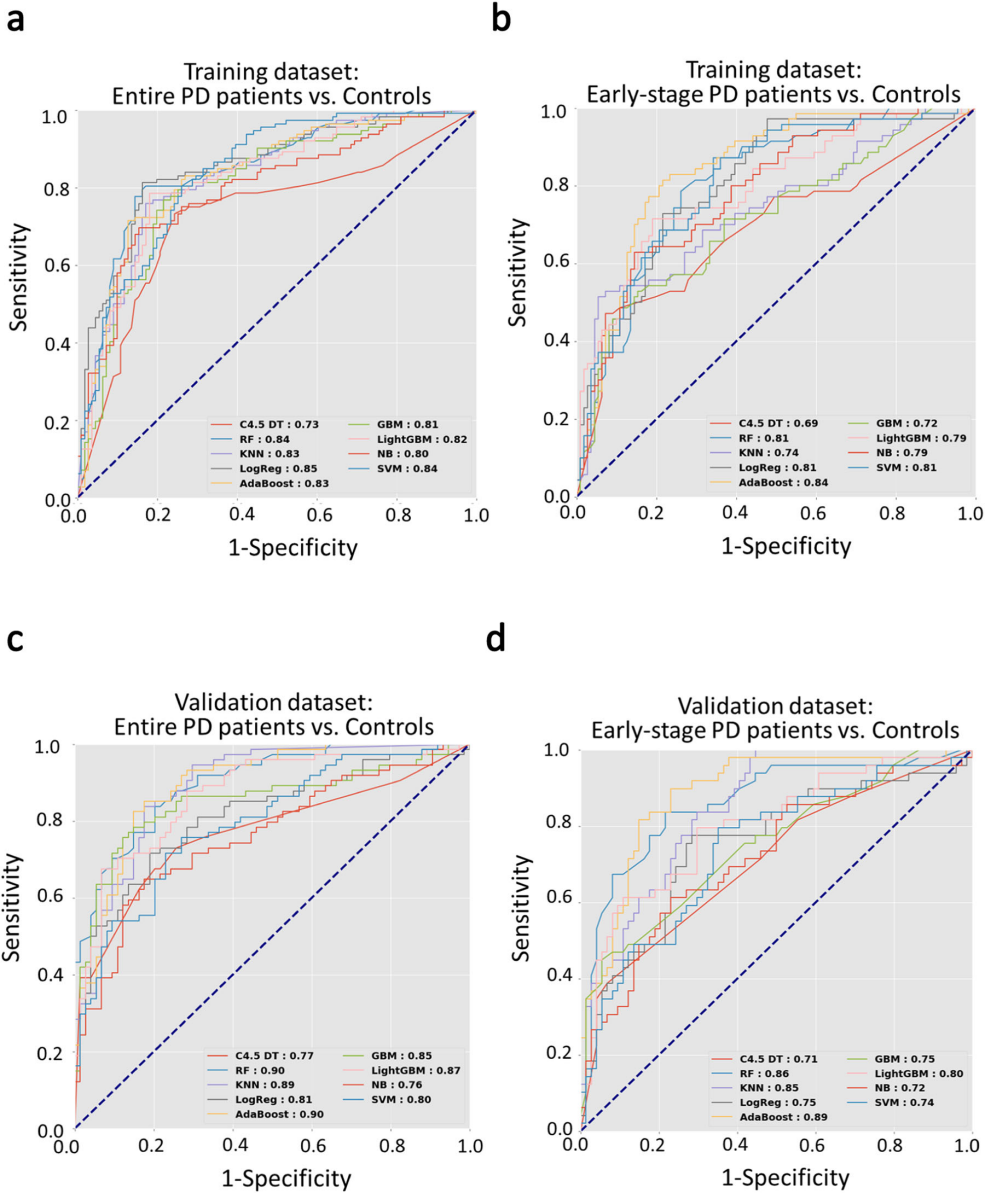
Receiver operating characteristic curves calculated with nine deep-learning classifier models, based on combined facial and voice biometric features. The models were tested for their ability to differentiate between **a** all patients with PD during the “on” phase and controls, and **b** patients with early-stage PD during the “on” phase and age-matched controls, in the training dataset. The models were confirmed with an independent validation dataset, where they differentiated between **c** all patients with PD during the “off” phase and controls, and **d** patients with early-stage PD during the “off” phase and age-matched controls.

Next, we further integrated the facial features, voice parameters, and basic characteristics of age and sex as an integrated model. We used the sequential forward selection method to select the best features for each classifier. The ROC analyses calculated with the logistic regression and random forest classifiers provided the optimal diagnostic values of 0.85 and 0.84, respectively, for distinguishing between entire patients with PD and controls, based on selected features of voice and facial expression characteristics (Fig. 2a; the selected features are shown in Supplementary Fig. 1A).

Given the high statistical significance of the above analysis, we assessed whether our artificial classifier, based on the deep-learning algorithm with integrated voice and facial expression features, might also be able to distinguish patients with early-stage PD during the “on” phase from age- and sex-matched controls. We found that the AdaBoost classifier achieved high diagnostic performance, with an AUROC of 0.84 (Fig. 2b; the selected features are shown in Supplementary Fig. 1B). The comparisons of diagnostic performance, accuracy scores, F1-scores, precision, and recall for all nine classifiers were shown in Supplementary Tables 3 and 4. The tables show the differentiation between all patients with diverse PD severities and controls and a separate analysis that focused on discriminating between patients with early-stage PD and matched controls.

### Diagnostic performance of the established model validated with a cohort with patients during “off” PD phase or drug naïve PD

Next, we validated the established model, which was based on the combined biometric features derived from patients with PD during the “on” phase. We tested whether this model could detect patients with PD that had not received anti-parkinsonism treatments to mimic real-world screening in community settings. This independent cohort included both patients in the “off” phase of PD and drug naïve patients with PD. In a ROC analysis, the model with the random forest classifier yielded an optimal diagnostic value of 0.90 for discriminating between patients with diverse PD severity and controls (Fig. 2c). To target patients with early-stage PD during the “off” state, we performed the ROC analysis with the AdaBoost classifier. This model had an optimal diagnostic value of 0.89 for discriminating between patients with early-stage PD and control participants (Fig. 2d).

## Discussion

In this study, we applied an integrated approach that included both voice and facial expression analyses combined with different deep-learning classifiers to discriminate between all patients with PD during the “on” phase and aged controls. The best model showed optimal diagnostic performance for distinguishing between patients with early-stage PD and age-matched controls. Furthermore, this model, which was based on selected voice and facial expression features, was validated with an independent cohort that comprised drug naïve patients or patients with PD during the “off” phase and another group of aged controls. This analysis mimicked the real-world situation in community screenings. Of note, the model showed high diagnostic performance for distinguishing between drug naïve patients or patients with early-stage PD during the “off” phase and normal controls. These results provided the evidence to show that voice and facial expressions analyzed with a deep-learning classifier could effectively discriminate between patients with early-stage PD and control individuals.

Hypomimia, or “poker” face, is considered a common feature among patients with PD^6^. In our study, a random forest classifier for analyzing the facial expressions of participants only showed a suboptimal diagnostic performance (AUROC = 0.69) for discriminating between all patients with PD and controls. This diagnostic performance was lower than the performance demonstrated in a previous study, where participants performed three facial mimicry tasks, including a smiling face, a disgusted face, and a surprised face; that model achieved a 95% classification accuracy^10^. In the present study, the participants did not perform facial muscle tasks; instead, the participants were recorded the natural facial expressions as they read an article during the voice recording. In this setting, we measured and analyzed the natural variance and movements in facial expressions and eye blinking. Among the individual facial expression features analyzed in the current study, not surprisingly, reduced eye blinking was the most crucial feature for distinguishing between the PD group and controls. Other facial movements, like mouth width or the mouth angle movement variance, were not remarkably different from controls during reading. Our observations supported findings in previous studies that showed facial expressions, even recorded in a natural way, could serve as a potential biometric marker for detecting PD^11^. Our results also supported the concept that a deep-learning algorithm that can analyze micro-expressions might assist physicians in identifying patients in the early stage of PD. Furthermore, we demonstrated that testing suitable classifiers on the facial features detected with automatic software analysis could identify subtle characteristics of facial expressions that distinguished patients with early-stage PD from controls.

Speech impairments, including articulation, phonation, prosody, and speech fluency, comprise another early feature of patients with PD^12^. A wide variety of speech or voice tasks have been employed for detecting speech impairments, including sustained vowel phonation^13^, syllable repetition tasks^14^, sentence repetition tasks^15^, and reading tasks^16^. One recent study investigated the speech impairments in 100 drug naïve PD patients and the same number of controls using a quantitative acoustic analysis of variable speech dimensions related to phonation, articulation, prosody, and speech timing^17^. The results identified an AUROC of 0.86 in men and 0.93 in women for discriminating PD patients from controls, which diagnostic performance is similar with our results. Furthermore, speech features combined with support vector machine classifier assisted detection of early-stage PD from controls with an accuracy of 89% in men and 70% in women, and provided an accuracy of 63% for detecting participants with REM-stage sleep behavior disorders (RBD) from controls^18^. These results combined with our findings support a recent multi-center European study that simple speech recordings with automatic speech analysis and machine learning classifier could serve as sensitive noninvasive early biomarkers for PD and those with the PD prodromal symptom of RBD^19^. The ability to speak is a unique, complex process, which can be subdivided into several dimensions, including respiration, phonation, articulation, and prosody^20^. Among these features, prosody is an important aspect of human verbal communication. Therefore, we did not adopt the commonly used single vowel phonation test; instead, we used an article reading task, which was previously used for assisting in the diagnosis of PD^16^. The reading task conveyed semantic, syntactic, and affective information; it also reflected the emotions of the speaker. We used objective acoustic analysis for the recorded voice and speech, and then digitized, processed, and analyzed the retrieved features with different deep-learning classifiers. This process comprised speech parameterization, consequent statistical analysis, and mathematical modeling. Our results revealed that the voice features alone showed an optimal diagnostic performance of 86%, based on the AdaBoost classifier. Previous evidence has shown that voice or speech impairments could be identified in a prodromal interval of 10 years before the PD diagnosis^21^, highlighting that voice parameters seem to be a suitable biometric feature for early detection of PD. Furthermore, most automatic voice condition analysis systems used for detecting PD are based on speech data recorded under acoustically controlled conditions. In contrast, our study was performed in a free-living real-world scenario, where the voice was recorded with a smartphone in a realistic acoustic environment. We showed that, under these conditions, we could differentiate between patients with PD, even early-stage PD, and controls. Recently, a speech analysis was performed to analyze the effect of PD on speech rhythm in two different speaking tasks, reading an article and spontaneous speech phenomena in the monologues, in a group of 20 PD patients and 20 healthy controls^22^. The results showed that there was no major difference in the speech rhythm of these two speaking tasks among patients with PD. Compared to read a simple script, the participants usually need more complex language planning process to perform a spontaneous speech. In this regard, the attention of the participant is mostly directed to the choice of words and the planning of what to say, which largely depend on the education levels, the personal working and lifestyle backgrounds. These factors may affect the fruitfulness of the spontaneous speech, which would broaden its diversity in the performance of acoustic analysis. The comparable performance in the acoustic analysis between reading task and spontaneous speech^22^ highlights the potential that it is possible to use the reading task alone to detect rhythmic differences between PD patients and controls, allowing future large-scale screening in the pooled database from various sources^23^. This model may also have the potential to be applied to stored voice database as a retrospective analysis to identify subjects who may have PD. Future longitudinal studies on larger PD cohorts and also on other languages, with different articulation and rhythmic patterns, are needed to support our observations.

The field of identifying PD in a pre-diagnostic phase is rapidly moving, with expanding momentum. Clinical and biofluid biomarkers are being developed to identify individuals in the earliest stages of PD. It is widely believed that mechanism-targeted neuroprotective drugs will have the highest likelihood of providing benefit in individuals that have not quite met the criteria for a PD diagnosis. According to the Movement Disorders Society (MDS), the best characterized prodromal markers of PD are early pre-motor features, including polysomnography-confirmed RBD, anosmia, depression, and constipation^24^. Along with these non-motor symptoms, subtle motor features may be present^25^. The clinical diagnosis of PD requires the presence of multiple motor features. Thus, when subtle or single motor abnormalities occur prior to diagnosis, along with early non-motor features, this period is best referred to as the pre-diagnostic phase of PD^25^. In this study, we identified that early PD related biometric features could be derived from facial expressions and voice parameters recorded during a reading task in a natural situation. With the aid of suitable deep-learning classifiers, an analysis of these features could assist in diagnosing PD in the early motor stage of the disease. Future studies incorporating more comprehensive biometric features, including gait analysis, and the MDS research criteria of characteristic prodromal markers of PD are needed to assist identifying PD patients at the earlier stage of the disease.

An advantage of our approach was the automatic acquisition of facial and voice features from smartphone video recordings. Electronic devices, such as smartphones, smartwatches, and tablets, contain several sensors that can acquire acoustic signals and facial expressions. This availability may enable low-cost screening in large populations where access to a neurologist is limited. Furthermore, the ability to access data remotely could empower neurologists to monitor patients effectively, objectively, and as frequently as necessary. Numerous studies have shown that information on motion activity could be harnessed with wearable sensors^26^. Advances in wearable technology and the availability of remote testing, combined with a widely available deep-learning algorithm, could aid in objective measurements of emerging subtle motor dysfunction in those at risk of PD. Indeed, our approach to speech and voice analyses could be combined with monitoring devices for detecting symptoms of RBD, gait, and motion. In the future, these techniques could be combined in an integrated multimodal biometric sensing platform for early PD detection and for monitoring PD progression.

Our study had several limitations. First, some patients with PD had jaw and voice tremors, but we did not subgroup these patients. Therefore, the inclusion of these patients might have partially affected the analyses of voice and speech. Second, we did not correlate the variance or features of speech and facial expressions with limb movement difficulties in patients with PD. However, impairments in speech prosody have not been clearly correlated with motor symptom scores, disease duration. or in particular, disease progression over time^27^. Third, we exclude PD patients with depression because the existence of depression has been shown to affect the speech features^28^, which would contribute to be a confounding factor in this analysis. However, depression is a common non-motor feature or even a prodromal feature of PD, future studies incorporating PD patients with or without depression would provide a more comprehensive picture to assist the early diagnosis of PD. Finally, we did not record serial acoustic or facial expression changes over time. Thus, we could not exclude the possibility that variability due to daily vocal and facial feature fluctuations could have influenced the results.

In conclusion, our results showed that the integrated biometric features of voice and facial expressions combined with deep-learning classifiers could assist the identification of early-stage PD patients from aged controls. In future, a multi-faceted biometric implemented in a longitudinal prospective study is needed to validate the potential of biometric markers in assisting with the early diagnosis of PD.

## Methods

### Subjects

We recruited 371 participants, including 186 patients with PD and 185 healthy controls, from National Taiwan University Hospital. PD was diagnosed according to the United Kingdom PD Society Brain Bank Clinical Diagnostic Criteria^29^. Controls were neurologically unaffected participants who were spouses or accompanying friends of the patients with PD. Participants underwent otolaryngologic evaluations, to exclude hearing loss and other non-neurologic disorders that might affect the vocal cords. They also underwent ophthalmologic evaluations, to exclude ophthalmologic disorders that could impair visual acuity. Participants were excluded when they were illiterate, had dementia (defined as a score <20 on the Montreal Cognitive Assessment scale)^30^, or had co-morbid depressive symptoms, assessed with the Movement Disorder Society-Unified Parkinson’s Disease Rating Scale (MDS-UPDRS), part I^31^. The severity of motor symptoms in patients with PD was evaluated with the MDS-UPDRS part III motor score^31^ and with Hoehn–Yahr staging^32^. Early-stage PD was defined as a Hoehn-Yahr stage <3, and advanced-staged PD was defined as a Hoehn-Yahr stage ≥3. All participants provided written informed consent and the institute of ethics board committee of National Taiwan University Hospital approved the study.

We grouped data from 186 patients with PD and 185 controls into a training set (112 PD patients during the “on” phase and 111 controls) and a validation set (74 patients during the “off” PD phase [*n* = 50] or drug-naïve patients with PD [*n* = 24] and 74 controls). The “on” and “off” phase were defined within 3 h and more than 12 h after the last dose of dopaminergic medication individually. The validation group assignment was based on whether an anti-parkinsonism medication effect was evident in patients with PD, when they underwent the voice and facial expression recordings.

Patients with idiopathic PD can respond well to levodopa therapy. In those cases, during the “on” phase, motor function may be similar to that observed in healthy aged individuals. Therefore, patients in the “on” phase of PD are more difficult to differentiate from controls than patients in the “off” phase of PD. Hence, we trained the model with the features derived during the “on“ phase that could differentiate PD patients from healthy controls. We reasoned that a model that could discriminate patients in the “on” phase of PD from controls might show optimal diagnostic performance in identifying drug-naïve patients with PD from healthy, aged individuals, in a real-world situation. Accordingly, the validation cohort was an independent cohort of patients in the “off” PD phase, which mimicked the drug naïve state. These patients were compared to an independent age- and sex-matched healthy control group.

### Experimental procedures

**Fig. 3:**
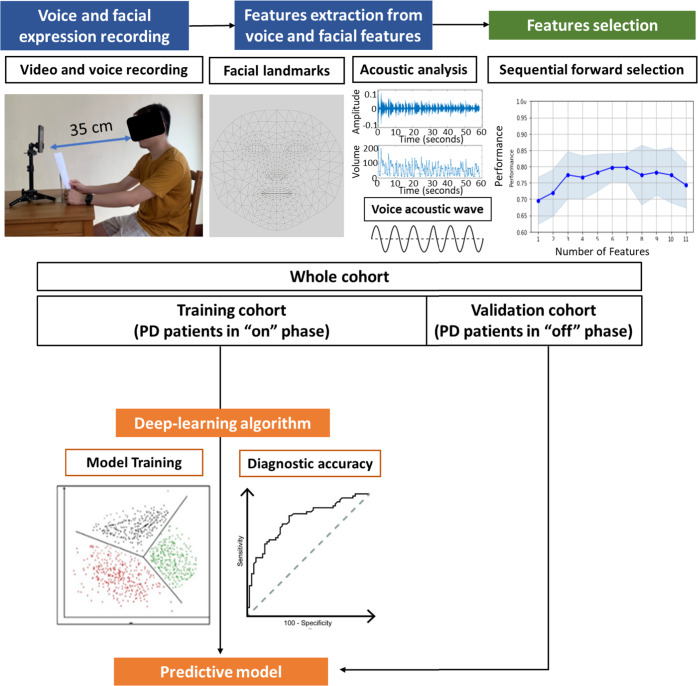
Schematic representation of the experimental paradigm. The written consent was obtained from the participant for publication of the photograph.

Both the script and the smartphone (iPhone 8 plus, Apple Inc.) were placed in front of the participant and the distance between the smartphone and the participant is 35 cm, which can record the voice and facial expression clearly (Fig. 3a). Experimental procedures are shown schematically in Fig. 3.

### Facial expression recordings and feature descriptions

Video recordings were performed by SJCAM SJ4000 (SJCAM Limited Co., Ltd.). Participants’ facial expressions were recorded when they were reading. Facial landmarks were extracted from these video recordings with Google MediaPipe Face Mesh (https://google.github.io/mediapipe/solutions/face_mesh.html).

**Fig. 4:**
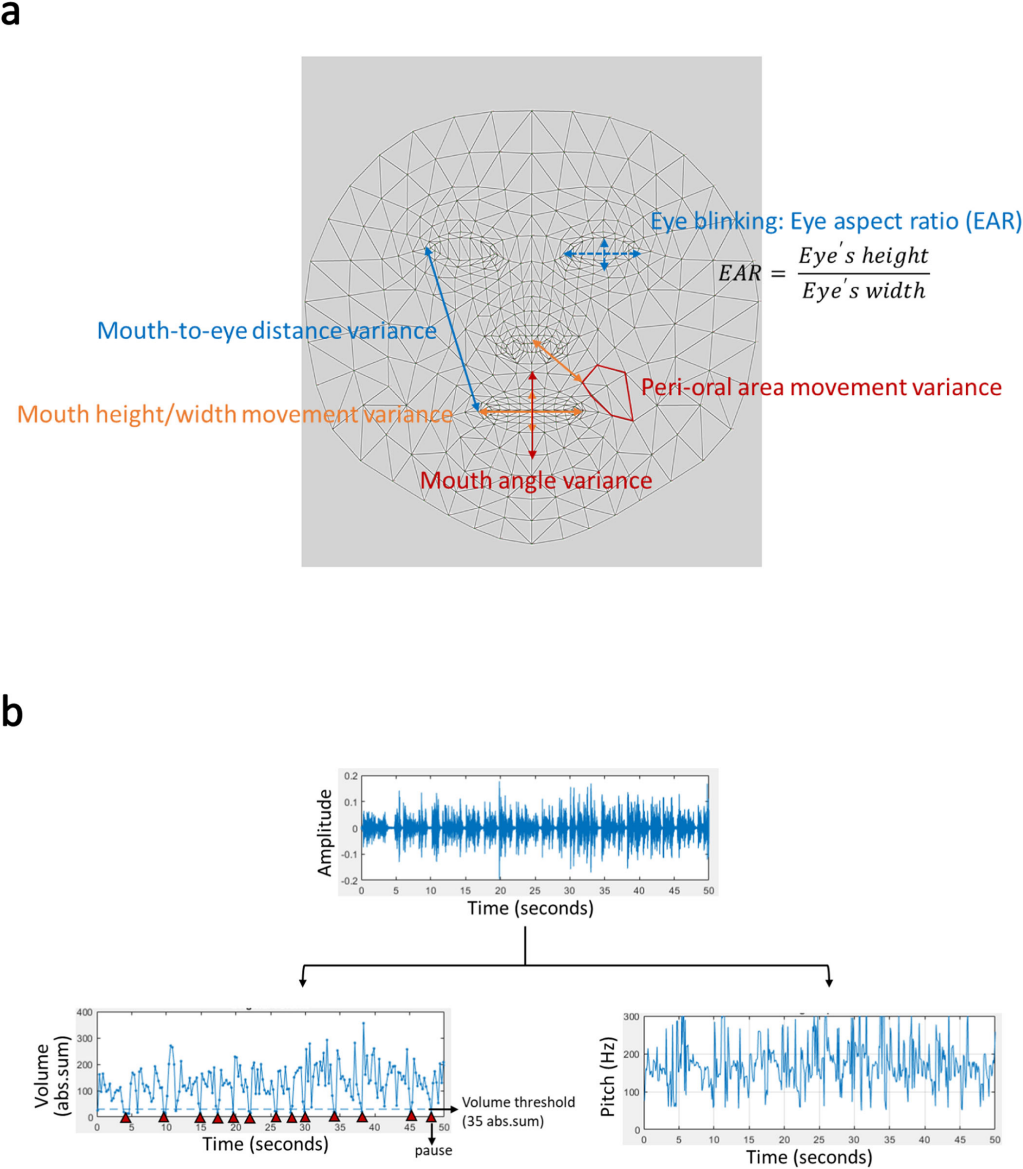
Voice and facial expression features analyzed in this study. **a** The diagram illustrates the facial features extracted from facial landmarks with Google Media Pipe Face Mesh. These features were: eye blinking (EAR), mouth to eye distance variance, mouth height and width movement variances, peri-oral area movement variance, and mouth angle variance. **b** The voice recording is divided into volume and pitch features.

Six key eye- and mouth-related features were calculated automatically based on the facial landmarks (Fig. 4a). These features were analyzed individually on both sides of the face. The eye-blinking feature was evaluated with various different rate thresholds. The mouth-related features included the mouth height/width variance; the mouth angle variance; the mouth-to-eye distance variances; and the peri-oral area movement variances. These features are defined below.

1. Eye blinking (with different thresholds): the total time spent blinking the eyes during the 30-s recordings. We applied the eye aspect ratio (EAR) to determine whether the eye had blinked. The EAR was calculated as the eye height divided by the eye width. We calculated the rolling average of EAR values within every 30 frames (1 s/frame). An eye blink was defined as a valley with a lower value than the overall EAR mean (thresholds: 30, 50, 70, and 90% of the mean value). Once the total eye blinking time was acquired, the value was divided by the total frame number.
2. Mouth to eye distance variance (right/left): we summed the changes between the current and previous frames in the distances from the eye corner landmark to the mouth corner landmark on each side. This sum was divided by the face width and the total frame number.
3. Mouth height variance: we summed the changes between the current and previous frames in the distances from the upper lip to the lower lip. This sum was divided by the face width and the total frame number.
4. Mouth width movement variance: we summed the changes between the current and previous frames in the distance between the mouth corners. This sum was divided by the face width and total frame number.
5. Mouth angle variance: we summed the changes between the current and previous frames in the mouth lip cross angle. The angle was calculated from two lines: a horizontal line drawn through both corners of the mouth (mouth width) and a vertical line drawn from the upper lip to the lower lip (mouth height). The sum was divided by the total frame number.
6. Peri-oral area movement variance (right/left): We selected 6 points near the mouth and jaw area. A distance was drawn to each point from the center of the nose. The distance change was calculated by subtracting the distance in the current frame from that in the previous frame, and dividing by the face width. We summed the distance changes for all six points, and divided by the total frame number.

The mouth to eye distance variance and the peri-oral area movement variance both had right and left values, and the eye blinking feature had four different thresholds. Therefore, we included 20 total features, including age and sex, in the model.

### Voice recordings and feature descriptions

Participants were asked to read an article containing 500 words. Voice samples were recorded in linear PCM format (.wav) at a sampling rate of 44.1 kHz with a 24-bit sample size. The signal was subsequently converted to 44.1-kHz and 16-bit in a linear PCM format.

For the voice analysis, we focused on volume and pitch features that might discriminate patients with PD from controls (Fig. 4b). Volume represents the loudness of the audio signal, which is correlated to the amplitude of the signals. Pitch represents the vibration rate of audio signals, which can be represented by the fundamental frequency, or equivalently, the reciprocal of the fundamental period of the voice audio signals. Six voice-related features were analyzed, including reading time, phonetic score, pause percentage, voice volume variance, average pitch, and pitch variance. These six voice features are defined below.

1. Reading time: the time between starting and finishing reading a paragraph.
2. Phonetic score: a phonetic score was given for every participant based on reading the paragraph. The phonetic scores were calculated (https://ss.mirlab.org/) with the acoustic model, and articulatory scores were calculated with the articulatory model.
3. Pause percentage: the percentage of time that a participant paused when he/she read the paragraph. First, we performed frame blocking, where a stream of audio signals was converted to a set of frames. The time duration of each frame was set at 25 milliseconds. For each participant, when the volume of a frame was lower than the threshold, the frame was counted as a pause. This threshold was smaller than the average volume of all audio signals. When the average volume of a participant was larger than 100, we set the threshold to 30. Otherwise, the threshold was set to 20.
4. Volume variance: the difference between the average volume of the first half of the audio signals and the average volume of the second half of audio signals, for each participant. The volume variance was computed as follows:
  1 $$\begin{array}{l}Volume\,variance = \\ \displaystyle\frac{{the\,average\,volume\,of\,the\,first\,half - the\,average\,volume\,of\,the\,second\,half}}{{the\,total\,average\,volume}}\end{array}$$
  A negative value indicated that the volume increased over time. In contrast, a small value indicated little change in the volume.
5. Average pitch: *Pitch* is an auditory sensation in which a listener assigns musical tones to relative positions on a musical scale, based primarily on their perception of the vibration frequency. For the pitch feature, we set the frame size to 10 ms. We created a pitch detection algorithm for audio signals by computing the number of zero-crossings during each frame. A zero-crossing is a point where the sign of a mathematical function changes (e.g., from positive to zero to negative or from negative to zero to positive). This term is commonly used in electronics, mathematics, and acoustics processing.
  To calculate the average pitch, we computed the average time spent on each audio signal over the total number of frames, as follows:
  2 $${{{\mathrm{Average}}}}\,{{{\mathrm{pitch}}}} = \frac{{\mathop {\sum}\nolimits_{t = 1}^n {CZ\left( t \right)} }}{n}$$
  where *n* is the total number of frames for an audio signal, and *CZ(t)* is the number of times that the signal crossed the 0 level reference (i.e., zero-crossing) during the time *(t)* of the frame.
6. Pitch variance: this feature was defined as follows:

3 $$Pitch\,variance = \frac{{\mathop {\sum}\nolimits_{r = 1}^n {\left| {CZ\left( r \right) - CZ\left( {r - 1} \right)} \right|} }}{n}$$

### Feature selection and machine-learning analysis

We applied the sequential forward feature selection algorithm to select the most salient features for differentiating between patients with PD and controls^33^. The best subset was selected by optimizing a specified performance metric, given an arbitrary classifier^34^. Multiple classifiers were applied, and each classifier was used as the base model in sequential forward selection. Features selected coverage percentage from each classifier was calculated and ranked.

We then used the following classifiers: C4.5 decision tree^35^, *k*-Nearest Neighbor^36,37^, support vector machine^38^, Naïve Bayes, random forest^39^, logistic regression, gradient boosting machine classifier^40^, AdaBoost^41^, and Light Gradient Boosting Machine^42^. These nine training classifiers were compared for their performance in terms of accuracy, precision, recall, F1-score, and the area under the receiver operating characteristic curve (AUROC) for binary classification. We used 10-fold cross-validation to obtain an objective estimate of performance. The source codes for all the classifiers are available in the science kits of application programming interface^43^.

### Statistical analysis

Continuous variables are expressed as the mean ± standard deviation. Categorical variables are expressed as numbers and percentages. We tested the homogeneity of variances with Levene’s test. Variables were compared with two-tailed *t* tests or analysis of variance (ANOVA), when normally distributed, or with the non-parametric t test, when assumptions of normality or homoscedasticity were violated. The diagnostic performance of the models was expressed as the AUROC, and 95% confidence interval (95% CI). All statistical analyses were performed with SAS (version 9.4, Cary, NC, USA) and Graphpad Prism (version 9.0.0, San Diego, California USA). *P*-values < 0.05 were considered statistically significant.

### Reporting summary

Further information on research design is available in the Nature Research Reporting Summary linked to this article.

## Supplementary information

Supplementary Tables and Figure

Reporting Summary

## Data Availability

The datasets generated during and/or analyzed during the current study are available from the corresponding author on request.
