# Supplementary material for: An integrated biometric voice and facial features for early detection of Parkinson’s disease
Source: NPJ Parkinsons Dis. 2022 Oct 29;8:145. doi: 10.1038/s41531-022-00414-8 (PMC9617232; doi:10.1038/s41531-022-00414-8)

Supplementary table 1. Clinical characteristics of study participants in the training dataset.

	Control, n=111	Early-stage PD, n=70		Advanced-stage PD, n=42		Controls vs. Early-PD	Early vs. Advanced-stage PD
		H-Y Stage 1, n=26	H-Y Stage 2, n=44	H-Y Stage 3, n=34	H-Y Stage 4, n=7	*P* value	*P* value
Gender, male, N (%)	55 (49.5)	13 (50.0)	23 (52.3)	21 (61.7)	4 (57.1)	0.10	0.06
Current age, years	68.2 ± 9.5	62.7 ± 11.6	67.7 ± 9.2	70.6 ± 7.6	74.8 ± 9.9	0.09	<0.01**
MDS-UPDRS part III score (on)	N.A.	8.6 ± 3.2	15.7 ± 5.9	20.8 ± 6.5	25.6 ± 7.3	N.A.	<0.01**
Reading time, seconds	51.9 ± 13.4	53.5 ± 14.0	68.7 ± 31.6	74.6 ± 36.5	73.3 ± 15.9	<0.01**	<0.01**
Phonetic score	96.5 ± 1.7	95.7 ± 2.8	96.2 ± 1.9	93.7 ± 5.1	93.0 ± 5.4	0.10	0.03*
Pause percentage	13.8 ± 7.2	19.8 ± 9.2	25.4 ± 12.3	29.4 ± 12.7	36.4 ± 11.8	<0.01**	<0.01**
Volume variance	-2.3 ± 10.4	-3.8 ± 13.3	-2.6 ± 11.3	-2.3 ± 10.4	-1.9 ± 12.8	0.11	0.87
Pitch variance	16.2 ± 4.1	12.6 ± 4.3	12.6 ± 4.4	11.6 ± 3.4	13.6 ± 5.9	<0.01**	<0.01**
Average pitch	154.5 ± 31.6	150.0 ± 29.8	150.9 ± 30.7	153.0 ± 34.4	168.3 ± 20.4	0.92	0.21
Mouth to eye distances variance (right)	0.004±0.002	0.004 ± 0.002	0.004 ± 0.002	0.003 ± 0.002	0.005 ± 0.002	0.32	0.41
Mouth to eye distances variance (left)	0.004±0.002	0.004±0.0028	0.005 ± 0.003	0.004 ± 0.003	0.005 ± 0.001	0.89	0.72

Mouth height variance	0.008±0.004	0.007±0.005	0.008±0.004	0.006±0.003	0.008±0.005	0.78	0.81
Mouth width variance	0.007±0.005	0.008±0.005	0.009±0.005	0.007±0.004	0.0088±0.0023	0.62	0.57
Mouth angle variance	0.881±0.432	0.892±0.367	0.976±0.612	1.010±0.612	1.211±1.072	0.16	0.15
Peri-oral area movement variance (right)	0.029±0.027	0.030±0.041	0.031±0.023	0.029±0.011	0.031±0.019	0.52	0.61
Peri-oral area movement variance (left)	0.030±0.022	0.031±0.023	0.032±0.024	0.032±0.025	0.038±0.029	0.92	0.78
Eye blinking (30% threshold)	0.1553±0.1582	0.1077±0.1406	0.1015±0.1470	0.1158±0.1946	0.1208±0.1976	<0.01**	<0.01**
Eye blinking (50% threshold)	0.0691±0.1096	0.0423±0.0585	0.0485±0.1000	0.0357±0.0759	0.0125±0.0248	0.03*	0.01*
Eye blinking (70% threshold)	0.0138±0.0303	0.0103±0.0263	0.0167±0.0531	0.0066±0.0217	0.0000±0.0000	0.06	0.01**
Eye blinking (90% threshold)	0.005±0.003	0.004±0.002	0.003±0.0002	0.002±0.001	0.001±0.002	0.67	0.03*

PD, Parkinson’s disease; MDS-UPDRS, the Movement Disorder Society-Sponsored Revision of the Unified Parkinson's Disease Rating Scale; H-Y stage; Hoehn-Yahr stage.

Supplementary table 2. Clinical characteristics of study participants in the validation dataset.

	Control, n=74	Early-stage PD, n=49		Advanced-stage PD, n=25		Controls vs. Early-PD	Early vs. Advanced-stage PD
		H-Y Stage 1, n=18	H-Y Stage 2, n=31	H-Y Stage 3, n=24	H-Y Stage 4, n=2	*P* value	*P* value
Sex, male, N (%)	29 (39.2)	7 (38.9)	15 (48.4)	20 (83.3)	1 (50.0)	0.07	<0.01**
Current age, years	69.1 ± 8.1	65.8 ± 7.0	66.0 ± 7.8	70.1 ± 6.9	67.4 ± 3.7	0.10	0.08
MDS-UPDRS part III score (off)	N.A.	12.6 ± 4.8	19.2 ± 7.1	25.8 ± 8.2	31.3 ± 7.9	N.A.	<0.01**
Reading time, seconds	53.2 ± 14.1	50.0 ± 12.7	59.8 ± 22.7	76.3 ± 30.4	53.3 ± 13.03	0.03*	<0.01**
Phonetic score	96.5 ± 1.7	95.7 ± 2.8	96.2 ± 1.9	93.7 ± 5.1	93.0 ± 5.4	0.82	0.05
Pause percentage	13.5 ± 8.1	16.2 ± 8.7	21.5 ± 11.8	31.2 ± 12.9	35.2 ± 12.3	0.02*	<0.01**
Volume variance	-3.4 ± 9.2	-3.5 ± 10.8	-2.9 ± 11.2	-2.8 ± 9.4	-2.1 ± 10.2	0.57	0.03*
Pitch variance	16.1 ± 5.2	13.2 ± 5.2	12.8 ± 5.4	11.9 ± 4.2	12.6 ± 6.9	0.04*	0.02*
Average pitch	154.5 ± 31.6	150.0 ± 29.8	150.9 ± 30.7	153.0 ± 34.4	168.3 ± 20.4	0.89	0.21
Mouth to eye distances variance (right)	0.003±0.002	0.003 ± 0.001	0.004 ± 0.002	0.003 ± 0.001	0.004 ± 0.002	0.91	0.82
Mouth to eye distances variance (left)	0.003±0.002	0.004±0.0003	0.004 ± 0.003	0.003 ± 0.002	0.004 ± 0.001	0.87	0.92

Mouth height variance	0.008±0.005	0.007±0.006	0.007±0.005	0.007±0.005	0.008±0.021	0.95	0.83
Mouth width variance	0.007±0.003	0.007±0.004	0.008±0.004	0.008±0.006	0.008±0.0027	0.91	0.43
Mouth angle variance	0.915±0.528	0.892±0.412	0.981±0.534	0.993±0.572	1.012±0.383	0.12	0.09
Peri-oral area movement variance (right)	0.030±0.025	0.031±0.025	0.032±0.071	0.030±0.031	0.033±0.021	0.52	0.61
Peri-oral area movement variance (left)	0.030±0.018	0.030±0.082	0.032±0.054	0.031±0.091	0.036±0.057	0.85	0.62
Eye blinking (30% threshold)	0.2271±0.1034	0.1723±0.1013	0.1521±0.1231	0.1248±0.1356	0.1301±0.1956	<0.01**	<0.01**
Eye blinking (50% threshold)	0.0721±0.1241	0.0531±0.0243	0.0412±0.0672	0.0323±0.0653	0.0122±0.0307	0.03*	0<0.01**
Eye blinking (70% threshold)	0.0142±0.0512	0.0112±0.0232	0.0169±0.0623	0.0073±0.0421	0.0048±0.0091	0.06	0.01**
Eye blinking (90% threshold)	0.006±0.004	0.004±0.003	0.004±0.0002	0.003±0.001	0.002±0.001	0.67	0.02*

PD, Parkinson's disease; MDS-UPDRS, the Movement Disorder Society-Sponsored Revision of the Unified Parkinson's Disease Rating Scale; H-Y stage; Hoehn-Yahr stage.

Supplementary table 3. Cross-validation metrics obtained from different classifiers for differentiating entire PD patients from elderly controls in the training dataset

Classifiers	AUROC, %	F1, %	Accuracy, %	Precision, %	Recall, %
C4.5 DT	72.92	73.06	73.54	74.77	71.43
RF	84.43	78.67	79.82	83.84	74.11
KNN	83.02	78.14	78.92	81.55	75.00
LogReg	85.37	81.82	82.06	83.33	80.36
AdaBoost	83.29	77.07	78.92	84.95	70.54
GBM	80.69	76.92	77.13	77.98	75.89
Light GBM	82.42	79.45	79.82	81.31	77.68
NB	79.91	74.76	76.68	81.91	68.75
SVM	83.90	77.39	76.68	75.42	79.46

AUROC, the area under the receiver operating characteristic; DT, C4.5 decision tree; KNN, k-Nearest Neighbor; SVM, support vector machine; NB, Naïve Bayes; RF, random forest; LogReg, logistic regression; GBM, gradient boosting machine classifier.

Supplementary table 4. Cross-validation metrics obtained from different classifiers for differentiating early-stage PD patients from elderly controls in the training dataset

	AUROC, %	F1, %	Accuracy, %	Precision, %	Recall, %
C4.5 DT	69.09	54.21	72.93	78.38	41.43
RF	80.93	71.43	73.48	61.22	85.71
KNN	73.96	63.06	77.35	85.37	50.00
LogReg	80.98	69.44	75.69	67.57	71.43
AdaBoost	84.39	74.67	79.01	70.00	80.00
GBM	71.77	57.39	72.93	73.33	47.14
Light GBM	79.43	70.00	76.80	70.00	70.00
NB	78.69	66.67	76.24	72.88	61.43
SVM	80.98	70.59	72.38	60.00	85.71

AUROC, the area under the receiver operating characteristic; DT, C4.5 decision tree; KNN, k-Nearest Neighbor; SVM, support vector machine; NB, Naïve Bayes; RF, random forest; LogReg, logistic regression; GBM, gradient boosting machine classifier.

Supplementary Figure 1. The sequential forward feature selection (SFS) algorithm was applied to obtain the most crucial features for differentiation. The selected features were used to differentiate between (A) all patients with PD during the "on" phase and all controls, and (B) patients with early-stage PD during the "on" phase and age-matched controls, in the training dataset.

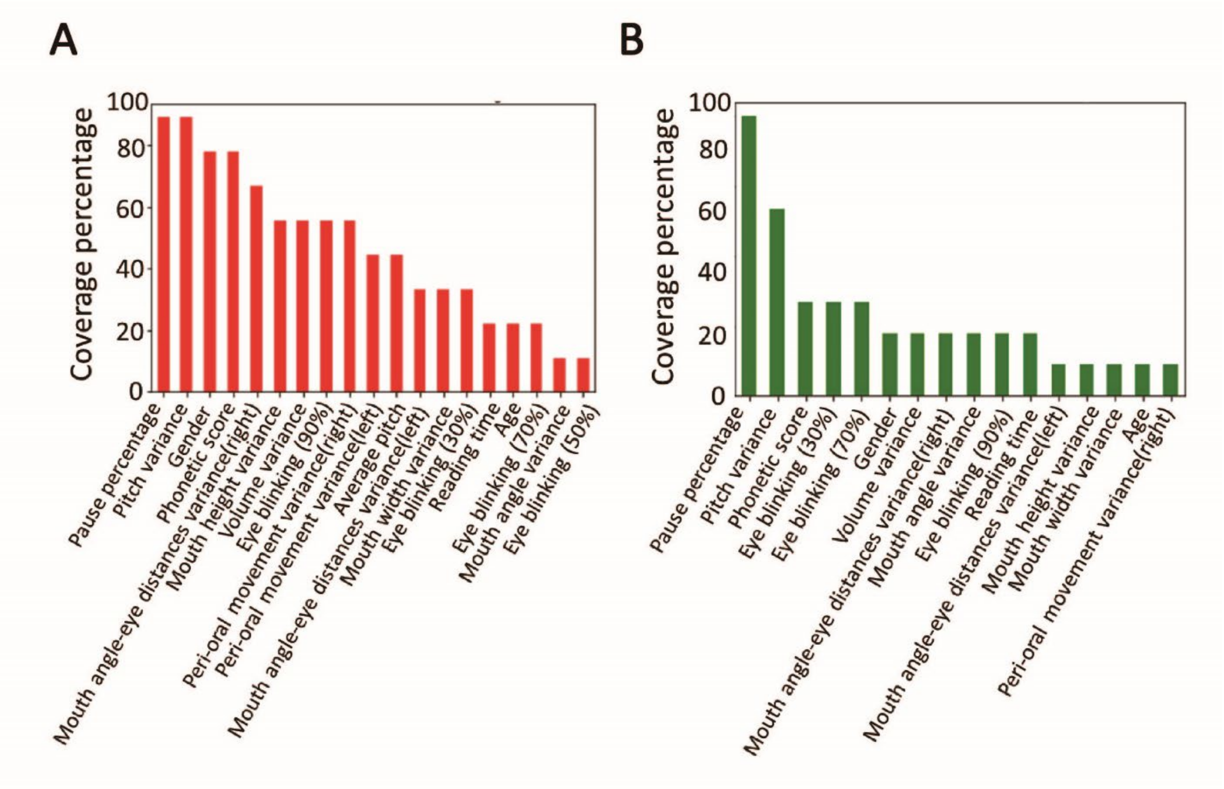

Supplement: Supplementary file 1 — Supplementary Tables and Figure [file 41531_2022_414_MOESM1_ESM.pdf]
