# Supplementary material for: An integrated biometric voice and facial features for early detection of Parkinson’s disease
Source: NPJ Parkinsons Dis. 2022 Oct 29;8:145. doi: 10.1038/s41531-022-00414-8 (PMC9617232; doi:10.1038/s41531-022-00414-8)
Supplement: Supplementary file 2 — Reporting Summary [file 41531_2022_414_MOESM2_ESM.pdf]

nature portfolio

Corresponding author(s): Chin-Hsien Lin

Last updated by author(s): Oct 7, 2022

Reporting Summary

Nature Portfolio wishes to improve the reproducibility of the work that we publish. This form provides structure for consistency and transparency in reporting. For further information on Nature Portfolio policies, see our Editorial Policies and the Editorial Policy Checklist.

Statistics

For all statistical analyses, confirm that the following items are present in the figure legend, table legend, main text, or Methods section.

n/a	Confirmed	
☐	☒	The exact sample size (n) for each experimental group/condition, given as a discrete number and unit of measurement
☐	☒	A statement on whether measurements were taken from distinct samples or whether the same sample was measured repeatedly
☐	☒	The statistical test(s) used AND whether they are one- or two-sided<br>*Only common tests should be described solely by name; describe more complex techniques in the Methods section.*
☐	☒	A description of all covariates tested
☐	☒	A description of any assumptions or corrections, such as tests of normality and adjustment for multiple comparisons
☐	☒	A full description of the statistical parameters including central tendency (e.g. means) or other basic estimates (e.g. regression coefficient) AND variation (e.g. standard deviation) or associated estimates of uncertainty (e.g. confidence intervals)
☒	☐	For null hypothesis testing, the test statistic (e.g. F, t, r) with confidence intervals, effect sizes, degrees of freedom and P value noted<br>*Give P values as exact values whenever suitable.*
☒	☐	For Bayesian analysis, information on the choice of priors and Markov chain Monte Carlo settings
☐	☒	For hierarchical and complex designs, identification of the appropriate level for tests and full reporting of outcomes
☒	☐	Estimates of effect sizes (e.g. Cohen's d, Pearson's r), indicating how they were calculated

Our web collection on statistics for biologists contains articles on many of the points above.

Software and code

Policy information about availability of computer code

Data collection	N.A.
Data analysis	N.A.

For manuscripts utilizing custom algorithms or software that are central to the research but not yet described in published literature, software must be made available to editors and reviewers. We strongly encourage code deposition in a community repository (e.g. GitHub). See the Nature Portfolio guidelines for submitting code & software for further information.

Data

Policy information about availability of data

All manuscripts must include a data availability statement. This statement should provide the following information, where applicable:

- Accession codes, unique identifiers, or web links for publicly available datasets
- A description of any restrictions on data availability
- For clinical datasets or third party data, please ensure that the statement adheres to our policy

The datasets generated during and/or analyzed during the current study are available from the corresponding author on request.

Human research participants

Policy information about studies involving human research participants and Sex and Gender in Research.

Reporting on sex and gender	We report the data of biological sex in table 1 and the supplementary tables.
Population characteristics	A population of 371 participants, including 186 patients with Parkinson's disease (PD) and 185 non-affected controls, was included in the study.
Recruitment	We recruited 371 participants, including 186 patients with PD and 185 healthy controls, from National Taiwan University Hospital. PD was diagnosed according to the United Kingdom PD Society Brain Bank Clinical Diagnostic Criteria. Controls were neurologically unaffected participants who were spouses or accompanying friends of the patients with PD.
Ethics oversight	All participants provided written informed consent and the institute of ethics board committee of National Taiwan University Hospital approved the study.

Note that full information on the approval of the study protocol must also be provided in the manuscript.

Field-specific reporting

Please select the one below that is the best fit for your research. If you are not sure, read the appropriate sections before making your selection.

☒ Life sciences ☐ Behavioural & social sciences ☐ Ecological, evolutionary & environmental sciences

For a reference copy of the document with all sections, see nature.com/documents/nr-reporting-summary-flat.pdf

Life sciences study design

All studies must disclose on these points even when the disclosure is negative.

Sample size	Sample size was determined based on the assumption of 5% of type I error to reach a minimum power of 80%.
Data exclusions	Participants were excluded when they were illiterate, had dementia (defined as a score <20 on the Montreal Cognitive Assessment scale), or had co-morbid depressive symptoms, assessed with the Movement Disorder Society-Unified Parkinson's Disease Rating Scale (MDS-UPDRS), part I.
Replication	We have resigned a validation cohort in the study to replicate the findings obtained from the training cohort.
Randomization	N.A.
Blinding	N.A.

Reporting for specific materials, systems and methods

We require information from authors about some types of materials, experimental systems and methods used in many studies. Here, indicate whether each material, system or method listed is relevant to your study. If you are not sure if a list item applies to your research, read the appropriate section before selecting a response.

Materials & experimental systems

n/a	Involved in the study	
☒	☐	Antibodies
☒	☐	Eukaryotic cell lines
☒	☐	Palaeontology and archaeology
☒	☐	Animals and other organisms
☐	☒	Clinical data
☒	☐	Dual use research of concern

Methods

n/a	Involved in the study	
☒	☐	ChIP-seq
☒	☐	Flow cytometry
☒	☐	MRI-based neuroimaging

Clinical data

Policy information about clinical studies

All manuscripts should comply with the ICMJE guidelines for publication of clinical research and a completed CONSORT checklist must be included with all submissions.

Clinical trial registration	N.A (This study is not a clinical trial).
Study protocol	The study protocol was described in details in the methods.
Data collection	The process of data collection was described in details in the methods.
Outcomes	The outcome measurement was described in details in the methods.
